# Differences in smoking behaviors and readiness to change for patients with COPD and differing categories of depressive symptoms: a descriptive cross-sectional design

**DOI:** 10.1186/s12890-023-02621-2

**Published:** 2023-09-08

**Authors:** Jessica Floyd, Jennifer Mallow, Kesheng Wang, Stephen M. Davis, Roger Carpenter, Laurie Theeke

**Affiliations:** 1 Waynesburg University, Waynesburg, PA USA; 2 West Virginia University, Morgantown, West Virginia USA; 3 The George Washington University, Washington, D.C. USA

**Keywords:** Chronic obstructive pulmonary disease, COPD, Depression, Smoking, Smoking cessation

## Abstract

**Background:**

Chronic obstructive pulmonary disease (COPD) is the third leading cause of death in the world with nearly 90% of cases caused by tobacco smoking. Nearly 40% of people with COPD are diagnosed with depression which impacts quality of life and smoking cessation. The purpose of this study was to describe factors influencing smoking behaviors and readiness to change in people with comorbid COPD and depression.

**Methods:**

A descriptive cross-sectional design was used. A convenience sample of 222 participants self-reported and/or had a documented diagnosis of COPD. Participants completed study measures which included the PHQ-9 for depressive symptoms, assessment of smoking behaviors using The Cigarette Dependence Scale, report of readiness to change using The Smoking Stage of Change Questionnaire, The Smoking Decisional Balance Questionnaire, and The Processes of Change Questionnaire. Electronic and paper questionnaires were used. Data was stored in RedCap and analyzed using SPSS version 26. Based on variable type, descriptive and comparative analyses were conducted using ANOVA, t-test, chi-square, Pearson correlation, linear regression, and multiple linear regression to determine the relationships between smoking behaviors, COPD, and depressive symptoms.

**Results:**

Only 18 participants were classified as having no depressive symptoms. Participants who smoked had high nicotine dependence and wanted to quit smoking. Overall, participants saw more cons to smoking and were engaged in the processes of change. The majority of participants were in the maintenance or contemplation stage. Cigarette dependence could decrease by 9% if depressive symptoms are treated.

**Conclusions:**

There is a need to assess COPD patients for depression and to assess COPD patients’ smoking behaviors and readiness to change. Adequate treatment of depression could promote an individual to move through the stages of change from chronic contemplation to action, thus improving smoking cessation efforts for individuals with COPD. Understanding patients’ smoking behaviors and readiness to change can aid in developing personalized interventions to achieve smoking cessation and improve long-term outcomes.

## Introduction

Currently, over 30.8 million Americans smoke cigarettes and over 16 million suffer from smoking-related diseases [1]. In the United States, more than 16.4 million people have COPD and tobacco causes 90% of COPD cases [2]. Despite the physical consequences of smoking, it is often the psychological impact of smoking that makes it difficult to achieve cessation. Being a smoker doubles an individual’s risk for developing depression, and in heavy smokers, that risk quadruples [3]. In the COPD population, the prevalence of depression is estimated at 40%; however, depression is missed by practitioners in approximately 50% of patients [4]. It is known that psychological distress is more prevalent in the COPD population, in comparison to the general population [5]. For this reason, it is said that COPD and depression have a bidirectional relationship [6].

A knowledge gap exists when seeking to comprehensively understand physiological and psychological factors that serve as facilitators and/or barriers to achieving smoking cessation [7]. In comparison to other smokers, COPD patients are known to have increased difficulty achieving cessation [5]. One study found that out of 509 COPD participants, only a third (31.2%) reported using an evidence-based cessation intervention [5]. There is a need to identify smokers in vulnerable groups within the psychological and behavioral health population. Inequalitites between those with psychological and behavioral health diagnoses and the general population will continue to grow, unless disparities in smoking prevalence are identified [8].

To improve smoking cessation outcomes, understanding contributing factors is necessary to guide intervention development that targets smoking behaviors concurrently with psychological and behavioral health symptoms [9]. The purpose of this study was to describe factors influencing smoking behaviors and readiness to change in people with comorbid COPD and depression.

## Methods

### Design

A descriptive cross-sectional design was used to meet the study aims to: (1) Describe the relationships between COPD, depressive symptoms, smoking behaviors, and readiness to change; (2) Analyze the relationships between COPD, depressive symptoms, smoking behaviors, and readiness to change.

### Sample

A convenience sample of participants were recruited from the rural Appalachian region using multifaceted and adaptable recruitment methods from February-November of 2020. Primary recruitment was first conducted face-to-face at a Primary Care Clinic. If patients met the inclusion criteria and elected to participate, they completed the survey via a tablet. Due to the COVID-19 pandemic, face-to-face recruitment occurred for one month and then was suspended. During the pandemic, a study mailing was conducted from a list of COPD patients provided by the Primary Care Clinic. Patients received the study flyer and cover letter. Patients then contacted the researcher to participate in the study. Other recruitment strategies during this time included study advertisement through the newspaper, local and statewide clinics, and the local hospital.

Once permitted, face-to-face recruitment resumed and was conducted at a Pulmonary Clinic. COPD patients were identified by the providers, and then the researcher approached the patients after their appointment. If patients met the inclusion criteria and elected to participate, they provided the researcher with their home address or email address, depending on their preference for completing the survey. A second mailing was also conducted from a list of COPD patients provided by the Pulmonary Clinic. For the second mailing, the patients received a paper copy of the survey with a prepaid return envelope. If patients met the inclusion criteria and elected to participate, they completed the survey and mailed it back to the researcher.

G*Power software version 3.0.10 was used to calculate that a sample size of 130 participants was needed to achieve adequate power for the planned analyses based on an effect size d = 0.5 (medium), α = 0.05, power = 1-*β* = 0.8 [10, 11]. Participants were included in the study if they were adults greater than 18 years of age, self-reported the ability to read English at a 6^th^ grade level, self-reported being a current or former smoker, and self-reported and/or had a documented diagnosis of COPD.

### Ethical conduct of research

A letter of approval for this study was obtained from the West Virginia University’s Institutional Review Board (IRB). The study was classified as exempt and did not require documentation of informed consent. Participation in the study was voluntary and prior to participating, potential participants read a cover letter and were informed of their right to withdraw from the study at any point. This study was a minimal risk study and participation did not interfere with clinical care. The study team had plans in place if survey questions triggered emotional responses that were concerning, and each participant was provided a referral list that included contact information for healthcare providers and the National Suicide Hotline. Participants received incentive in the form of a $10 gift card to Walmart.

### Theoretical framework

The Transtheoretical Model was the theoretical framework that guided this study. The Transtheoretical Model assesses the essence of the behavior, identifies the stage of change individuals are in, and then selects stage specific interventions that aid in promoting change. This approach is designed to personalize interventions and enhance personal self-control. The Transtheoretical Model includes five stages of change, ten processes of change, decisional balance, self-efficacy, and temptation [12]. Self-efficacy and temptation were not measured in this study.

### Study variables and instruments

#### General characteristics survey

Using a self-reported demographic questionnaire, participant characteristics collected included categorical education level, gender, income, race, and comorbid conditions. In addition, continuous age, self-reported diagnosis of depression (yes/no), and medications for depression (yes/no) were collected.

#### Depression

Depressive symptoms were assessed as a continuous variable using the Patient Health Questionnaire-9 (PHQ-9). The PHQ-9 is a 10-item questionnaire. Participants were asked, “Over the last two weeks, how often have you been bothered by any of the following problems?” Participants rated their responses from 0 (not at all) to 3 (nearly every day). The participants total score was then calculated, with a higher score indicating more severe depressive symptoms. A score of 1–4 indicated minimal depressive symptoms, a score of 5–9 indicated mild depressive symptoms, a score of 10–14 indicated moderate depressive symptoms, a score of 15–19 indicated moderately severe depressive symptoms, and a score of 20–27 indicated severe depressive symptoms. The PHQ-9 was not used as a diagnostic criteria for depression. The PHQ-9 is a valid instrument with Cronbach alphas of 0.86–0.89 and test–retest reliability of 0.84 [13].

#### Smoking behaviors

Smoking behaviors were conceptually defined as patients’ smoking status, frequency of use, and nicotine dependence. These smoking behaviors were measured using The Cigarette Dependence Scale (CDS-12). Participants only completed this tool if they were current smokers. The level of cigarette dependence varies from 12, the lowest level of dependence, to 60, the highest level of dependence. The CDS-12 is a valid and reliable tool with psychometric testing of test–retest correlation of  > 0.60 for all items and  > 0.83 overall. Internal consistency is high with Cronbach’s alpha  > 0.84 [14].

#### Readiness to change

Readiness to change was conceptually defined as the perceived stage, pros and cons, and situations and thoughts that occur in the processes of change that affect smoking habits. Readiness to change was derived from the Transtheoretical Model. The Smoking Stage of Change Questionnaire (SSCQ), The Smoking Decisional Balance Questionnaire (SDBQ), and The Processes of Change Questionnaire (PCQ) were used to operationalize readiness to change.

##### Smoking Stage of Change Questionnaire (SSCQ)

The perceived stage of change was measured by the SSCQ, which is a three-item instrument. This questionnaire works as an algorithm, and depending on responses, participants were categorized in a stage of change. If participants had quit smoking within the last six months, they were placed in the action stage. If participants had quit more than six months ago, they were placed in the maintenance stage. If participants were thinking about quitting in the next 30 days and had one 24-h quit attempt in the past year, they were placed in the preparation stage. If participants were thinking about quitting in the next 30 days and had no quit attempt in the past year, they were placed in the contemplation stage. If participants were thinking of quitting in the next six months, they were also placed in the contemplation stage. If participants were not thinking of quitting, they were placed in the precontemplation stage. There is no former psychometric data to support this instrument; however, this instrument was specifically derived from the concepts of the Transtheoretical Model.

##### Smoking Decisional Balance Questionnaire (SDBQ)

The pros and cons of smoking were measured by the SDBQ. The SDBQ is a 20-item instrument that uses a 5-point likert scale. Participants were asked to rate the importance each statement had on their decision to smoke. Selecting 1 indicated not important at all and selecting 5 indicated extremely important. Items were added and averaged to produce two subscales, pros of smoking and cons of smoking. The difference of the scores can be calculated by subtracting the cons of smoking from the pros of smoking. If the difference is positive, the participants saw more pros of smoking. If the difference is negative, the participants saw more cons of smoking. The SDBQ is a valid and reliable tool with Cronbach alphas of 0.87 (pros) and 0.90 (cons) [15].

##### The Processes of Change Questionnaire (PCQ)

The situations and thoughts that affect smoking habits were measured by PCQ. To progress from one stage to another, individuals use the following ten processes of change: consciousness raising, dramatic relief, self-reevaluation, environmental reevaluation, self-liberation, social liberation, counterconditioning, stimulus control, contingency/ reinforcement management, and helping relationships [12]. The PCQ is a 20-item instrument. Participants were asked to rate how often the listed experiences or events had happened to them in the last month. Participants rated their responses from 1 (never) to 5 (repeatedly). The sum of all the scores gives a mean processes of change score, with a higher score indicating engagement in the processes of change. The means of the processes of change subscales were also calculated. The PCQ is a valid and reliable tool, with Cronbach alphas of 0.69–0.92 for each of the 10 subscales [16].

### Data analysis

Deidentified data were analyzed using the Statistical Package for the Social Sciences (SPSS) 26. Preliminary analyses included comprehensive descriptive analysis to describe the study sample and questionnaire results. The categorical variables were presented as raw values and percentages, while continuous variables were presented as mean ± SD. One-way analysis of variance, chi-square test for independence, Pearson correlation, and t-tests were conducted to identify statistically significant covariates for the dependent variables.

#### Aim 1

Pearson correlation was used to describe the relationships between COPD, depressive symptom severity, and the CDS-12, SDBQ, and PCQ.

#### Aim 2

Linear regression was used to determine explanatory value for variables with significant associations. Based on the Pearson correlation results, the CDS-12 and the PCQ were used in the linear regression. Then a multiple linear regression was used to control for the statistically significant covariates that were identified from the descriptive analyses. Based on the analyses, the CDS-12 was analyzed while controlling for self-reported depression, and the PCQ was analyzed while controlling for self-reported depression, gender, and age.

## Results

### Descriptive results

The sample of 222 participants [108 males (48.6%), 112 females (50.5%] ranged in age from 32 to 94 years old (*m* = 64 years (SD = 9.6). Majority (95%) were white and over 50% lived with lower socioeconomic status. In this sample, 113 participants (50.9%) self-reported that they did not have depression, and 101 participants (45.5%) stated they did have depression. When asked if they took medications to treat depression, 133 participants (59.9%) reported no, and 79 participants (35.6%) reported yes. Depressive symptom severity ranged from 0–27, with a mean of 7.9 (SD = 6.4). For this sample, only 18 participants (8.1%) were classified as having no depressive symptoms. Descriptive statistics on general participant characteristics and frequencies for scores on the PHQ-9 are displayed in Table 1.

**Table 1:** Descriptive statistics: general characteristics survey/PHQ-9 results

Variable	N (%)
**Gender**	
Male	108 (48.6)
Female	112 (50.5)
**Income**	
< $24,000	111 (50)
$25,000-$49,999	56 (25.2)
$50,000-$74,999	25 (11.3)
$75,000-$99,999	11 (5)
> $100,000	10 (4.5)
**Race**	
White	211 (95)
Black	4 (1.8)
Hispanic	2 (0.9)
Other	1 (0.5)
**Education Level**	
No formal education	1 (0.5)
Completed elementary	3 (1.4)
Some high school	30 (13.5)
Completed high school	67 (30.2)
Some college	51 (23)
Associate degree	18 (8.1)
Bachelor’s degree	17 (7.7)
Master’s degree	7 (3.2)
Other	7 (3.2)
GED	18 (8.1)
**Comorbid Conditions**	
Myocardial infarction	17 (7.7)
Congestive heart failure	20 (9)
Peripheral vascular disease	18 (8.1)
Cerebral vascular accident/TIA	8 (3.6)
Peptic ulcer disease	6 (2.7)
Liver disease	12 (5.4)
Diabetes mellitus	32 (14.4)
Other	19 (8.6)
**Do you have depression?**	
No	113 (50.9)
Yes	101 (45.5)
**Do you take medications to treat depression?**	
No	133 (59.9)
Yes	79 (35.6)
**Severity of Depressive Symptoms**	
No depressive symptoms	18 (8.1)
Minimal depressive symptoms	59 (26.6)
Mild depressive symptoms	52 (23.4)
Moderate depressive symptoms	39 (17.6)
Moderately severe depressive symptoms	25 (11.3)
Severe depressive symptoms	12 (5.4)

*Abbreviations*: *GED* refers to general education development, *TIA* refers to transient ischemic attack

Severity of Depressive Symptoms results are from the PHQ-9

Scores for the CDS-12 ranged from 23–54 with a mean score of 43.82. Scores for the PCQ ranged from 19.05–88.2 with a mean score of 45.8. Scores on the SDBQ ranged from -30.4–21.4 with a mean score of -5.78. For the PCQ sub-scales, participants used more experiential processes (23.65) than behavioral processes (20.4). Social liberation was the most used process of change (4.72), and stimulus control was the least used (2.56). Table 2 presents mean scores for the CDS-12, SDBQ, and PCQ (including subscales).

**Table 2:** Descriptive statistics: CDS-12, SDBQ, PCQ, PCQ sub-scales

Variable	M	Range	SD
Cigarette Dependence Scale	43.8	23.0–54.0	8.0
Smoking Decisional Balance Questionnaire	-5.8	-30.4–21.4	10.8
Processes of Change Questionnaire	45.8	19.1–88.2	15.9
- Behavioral process	20.4	9.1–42.2	8.0
- Experiential process	23.7	9.1–43.3	8.0
- Consciousness raising	4.3	1.5–7.5	1.8
- Dramatic relief	3.5	1.5–7.5	1.7
- Environmental reevaluation	3.5	1.5–7.5	2.0
- Self-reevaluation	3.5	1.5–7.5	1.9
- Social liberation	4.7	1.5–7.5	1.8
- Contingency/reinforcement management	3.0	1.5–7.5	1.9
- Counter conditioning	3.4	1.5–7.5	1.7
- Helping relationships	3.7	1.5–7.5	2.0
- Self-liberation	4.1	1.5–7.5	2.0
- Stimulus control	2.6	1.5–7.5	1.6

The SSCQ results found that 87 participants (39.2%) reported they currently smoked, 4 participants (1.8%) reported they quit within the last 6 months, 115 participants (51.8%) reported they quit more than 6 months ago, and 2 participants (0.9%) reported they never smoked. When asked if those who smoked were thinking of quitting, 25 participants (11.3%) reported they wanted to quit within the next 30 days, 32 participants (14.4%) reported they wanted to quit within the next 6 months, and 28 participants (12.6%) reported they were not thinking of quitting.

The SSCQ was also used to classify participants into their perceived stage of change. In this study, 28 (12.6%) participants were in the precontemplation stage; 39 (17.6%) participants were in the contemplation stage; 17 (7.7%) participants were in the preparation stage; 4 participants (1.8%) were in the action stage; and 115 (51.8%) participants were in the maintenance stage. Table 3 presents descriptive information about current smoking and descriptive information about participants’ current stage of change.

**Table 3:** Descriptive statistics: SSCQ

Smoking stage of change	N (%)
**Are you a current smoker?**	
Yes, I currently smoke	87 (39.2)
No, I quit within the last 6 months	4 (1.8)
No, I quit more than 6 months ago	115 (51.8)
No, I have never smoked	2 (0.9)
**Are you seriously thinking of quitting smoking?**	
Yes, within the next 30 days	25 (11.3)
Yes, within the next 6 months	32 (14.4)
No, not thinking of quitting	28 (12.6)
**Smoking Stage of Change Results**	
Precontemplation	28 (12.6)
Contemplation	39 (17.6)
Preparation	17 (7.7)
Action	4 (1.8)
Maintenance	115 (51.8)

#### Statistically significant covariates for the dependent variables

Differences in the CDS-12 were sought based on self-reported depression. There was a statistically significant difference in scores for self-reported depression, “Yes, I have depression” (M = 46.0, SD = 7.6) and “No, I do not have depression” (M = 41.6, SD = 7.9; t (72) = 2.5, *p* = 0.0, two tailed).

Differences in the PCQ were sought based on self-reported depression. There was a statistically significant difference in scores for self-reported depression, “Yes, I have depression” (M = 49.2, SD = 14.3) and “No, I do not have depression” (M = 42.8, SD = 17.0; t (131) = 2.4, *p* = 0.0, two tailed).

Differences in the PCQ were sought based on gender. There was a statistically significant difference in scores for gender, males (M = 41.4, SD = 13.8) and females (M = 50.4, SD = 16.7; t (134) = -3.4, *p* = 0.0, two tailed).

There was a statistically significant small, negative inverse correlation between age the PCQ, *r* = -0.2, *n* = 135, *p* = 0.0.

### Aim 1 Results

#### Pearson correlation analysis

There was a statistically significant medium, positive correlation between COPD, depressive symptoms, and the CDS-12, *r* = 0.3, *n* = 74, *p* = 0.0, with high levels of cigarette dependence associated with more severe depressive symptoms. There was a small, non-significant negative correlation between COPD, depressive symptoms, and the SDBQ, *r* = -0.0, *n* = 121, *p* = 0.9, with high levels of depressive symptoms associated with the patient endorsing more cons for smoking. There was a statistically significant small, positive correlation between COPD, depressive symptoms, and the PCQ, *r* = 0.3, *n* = 132, *p* = 0.0, with high use of the processes of change associated with more severe depressive symptoms.

### Aim 2 Results

#### Linear regression analysis

The ability of one control measure (depressive symptom severity) to predict cigarette dependence (CDS-12) was assessed. The total variance explained by the model was 9%, F (1, 72) = 8.2, *p* < 0.0.

The ability of one control measure (depressive symptom severity) to predict processes of change (PCQ) was assessed. The total variance explained by the model was 5.9%, F (1, 130) = 9.19, *p* < 0.00.

#### Multiple linear regression analysis

##### CDS-12- controlling for self-reported depression

Self-reported depression explained 10% of the variance in cigarette dependence (Table 4). No control measures were statistically significant, with self-reported depression recording a higher beta value (beta = 2.9, *p* = 0.2) than PHQ-9 (beta = 0.3, *p* = 0.1).

**Table 4:** Multiple linear regression: CDS-12 and depressive symptom severity- controlling for self-reported depression

Variable	B	SE	95% CI	*P*
PHQ-9	0.3	0.2	-0.1–0.6	0.1
Self-reported depression	2.9	2.1	-1.3–7.2	0.2
**Variable**	**Mean**	**Standard deviation**		
**CDS-12**	**43.8**	**8.1**		
**PHQ-9**	**9.8**	**6.8**		
**Self-reported depression**	**0.5**	**0.5**		

##### PCQ- Controlling for self-reported depression, gender, and age

Self-reported depression explained 6.3% of the variance in processes of change (Table 5). No control measures were statistically significant, with self-reported depression recording a higher beta value (beta = 4.0, *p* = 0.3) than PHQ-9 (beta = 0.5, *p* = 0.1). Gender explained 12% of the variance in processes of change (Table 5). Two control measures were statistically significant, with gender recording a higher beta value (beta = 8.3, *p* = 0.0) than PHQ-9 (beta = 0.5, *p* = 0.0). Age explained 7.1% of the variance in processes of change (Table 5). One control measure was statistically significant, with the PHQ-9 recording a higher beta value (beta = 0.5, *p* = 0.0) than age (beta = -0.2, *p* = 0.1).

**Table 5:** Multiple linear regression: PCQ and depressive symptom severity- controlling for self-reported depression, gender, and age

Variable	B	SE	95% CI	P
PHQ-9	0.5	0.3	-0.1-1.0	0.1
Self-reported depression	4.0	3.4	-2.8-10.7	0.3
PHQ-9	0.5	0.2	0.1-0.9	0.0
Gender	8.3	2.7	3.0-13.6	0.0
PHQ-9	0.5	0.2	0.1-1.0	0.0
Age	-0.2	0.2	-0.5-0.1	0.1
**Variable**	**Mean**	**Standard deviation**		
**PCQ**	**45.8**	**16.1**		
**PHQ-9**	**8.7**	**6.5**		
**Self-reported depression**	**0.5**	**0.5**		
PCQ	45.8	15.9		
PHQ-9	8.6	6.4		
Gender	1.5	0.5		
**PCQ**	**45.8**	**16**		
**PHQ-9**	**8.6**	**6.5**		
**Age**	**62.4**	**9.8**		

## Discussion

The characteristics of a convenience sample of people living with COPD and differing categories of depressive symptoms who sought care in West Virginia aligned with the demographics of the state. In the state, females have a higher prevalence of COPD at 16.6%, in comparison to males at 14% [17]. In West Virginia, 93.6% of the population is white, and COPD is found to be prevalent in West Virginia residents ages 55–64, who have an income of less than $15,000 [18].

The findings related to education in this study are characteristic of West Virginia residents. The vast majority of residents graduate from high school (86.9%), and few residents receive a college education (20.6%) [19]. West Virginia ranks first in the nation for cardiovascular disease and second in the nation for diabetes [18]. The percentage of adults that self-report depression in West Virginia is 26.4% [20]. In comparison to the self-reported depression findings of this study, this indicates that the prevalence of depression is higher among a sample of patients with COPD (45.5%). These findings help identify sociodemographic factors that are characteristic for patients with comorbid COPD and depressive symptoms who seek care in West Virginia.

National and international studies report similar findings to this study. In a national sample, it is known that COPD is more prevalent in individuals that have a lower income, and psychological distress is reported in 42.8% of COPD patients [17]. In a national sample of patients that self-reported COPD, the majority of participants were white women, 65 years and older; were of low income; and attended one year or more of college [21]. A study based in Germany found the prevalence of smoking in a COPD sample to be 38%. In comparison to this study, where current smokers made up 39.2% of the sample [5]. Using Beck’s depression inventory, a study in Spain reported that depression was more prevalent in a COPD sample in comparison to a control group [6]. These characteristics are also similar to those with COPD and depressive symptoms who sought care in West Virginia.

The findings related to COPD, depressive symptoms, and smoking behaviors are similar to previously published literature, with higher depressive symptom severity correlating with higher levels of cigarette dependence (CDS-12). It is well known that the prevalence of smoking is higher for those that have depression [9]. Those that have a psychological and behavioral health diagnosis are less likely to achieve cessation than the general population [22]. A dose–response relationship between smoking and mood disorders exists [23].

This was the first study to examine the relationship between COPD, depressive symptoms, and smoking behaviors with a sample of participants who sought care in West Virginia. The SDBQ findings indicated that the sample saw more negative aspects to smoking then positives. Participants perceptions on the pros and cons of smoking are important to evaluate, as they can vary depending on the stage of change. Cigarette dependence could decrease by 9% if depressive symptoms are treated. This emphasizes the importance of screening and treating patients with COPD for depression in clinical practice. When controlling for self-reported depression, it explained 10% of the variance in cigarette dependence. However, the self-reported depression findings were inaccurate, and this finding is not statistically significant. Given the PHQ-9 results, many participants did not know they were experiencing depressive symptoms and needed to be further assessed for diagnostic criteria for depression. This emphasizes the need to use validated depression screening tools in clinical practice.

For this study, high use of the processes of change (PCQ) was associated with more severe depressive symptoms. Participants with higher depressive symptom severity want to quit and want to progress through the stages of change. Focusing on depression symptom control may be the key to assisting patients through the stages of change and ultimately improving cessation rates and COPD outcomes. Depressive symptom severity explained 5.9% of the variance in the population’s engagement in the processes of change. Given the possible reciprocal relationship between depressive symptom severity and the population’s engagement in the processes of change, this finding could illustrate how participants experience chronic contemplation or behavioral procrastination.

The Transtheoretical Model states that uncertainty causes individuals to dwell in the contemplation stage [12]. Participants want to use the processes of change to progress through the stages of change; however, depressive symptoms interfere with their progression. When controlling for the variables, gender, age, and self-reported depression, the variances were analyzed. However, age and gender are not modifiable variables. Depressive symptom severity is the only variable that can be modified for this population to promote engagement in the processes of change. Knowing that the self-reported depression variable may not have captured true depression status, the use of validated depression screening tools is vital for this population.

### Limitations

Limitations of the study included the descriptive cross-sectional design, as a true cause and effect relationship cannot be determined [24]. A convenience sample was used to recruit participants, which is prone to sampling bias [24]. Several inclusion criteria were self-reported and were not otherwise validated. All of these factors could lead to a misrepresented sample. Generalizability of the results to other geographical locations is limited due to the racial homogeneity of the sample.

There is no formal psychometric data to support the SSCQ. A total PCQ score was used for data analysis. This score gives information regarding participants’ overall engagement in various processes of change. It does not analyze the specific processes of change being used by participants. Further analysis utilizing the PCQ sub-scales would be beneficial for future research.

Despite over sampling, this study was not adequately powered for all statistical analyses. Data that was received by a mailed survey was entered into RedCap by the researcher. Errors in data input may have occurred during this process. Data was also collected during the COVID-19 pandemic. For the COPD population, severity of depressive symptoms may have been impacted by the pandemic and the social isolation experienced during quarantine.

### Implications

Understanding the smoking behaviors of this population is important, as smoking behaviors impact disease management and outcomes. Depression and nicotine dependence could be factors that are impacting smoking behaviors and smoking cessation in this population. Interventions aimed at treating depression instead of solely on smoking cessation seem to be warranted. There is also a need for personalized interventions that target the stage of change an individual is in. Focusing on depression control may be the key to assisting patients through the stages of change and ultimately improving cessation rates and COPD outcomes.

Future research should be aimed at adequately assessing, diagnosing, and treating depression to determine the impact on outcomes, specifically related to smoking behaviors and readiness to change. Patients’ depression diagnoses should be compared with patients PHQ-9 results. Examining the rate of smoking cessation when depression is controlled for is warranted.

## Conclusion

Patients with comorbid COPD and depression require variability in treatment. Successful management of these patients includes understanding their sociodemographic factors, depression severity, smoking behaviors, and readiness to change. COPD patients need to receive continual screening and treatment for depression. Treatment for depression can improve COPD patient outcomes and aid in achieving smoking cessation. For current smokers, smoking behaviors need to be assessed to understand patients’ current level of depression, smoking status, frequency of use, and nicotine dependence. To achieve cessation, the relationship between depression and readiness to change needs to be assessed to develop personalized smoking cessation interventions. Practitioners must understand patients perceived stage, pros and cons, and situations and thoughts that occur in the process of change to help achieve cessation. The relationships between smoking, COPD, and depression must be recognized to address the complex health care needs of this population.

## Data Availability

The dataset used and/or analyzed during the current study are available from the corresponding author on reasonable request.

## Abbreviations

CDS-12: The cigarette dependence scale
PHQ-9: Patient health questionnaire-9
PCQ: The processes of change questionnaire
SDBQ: The smoking decisional balance questionnaire
SSCQ: The smoking stage of change questionnaire

## References

[1] Centers for Disease Control and Prevention. Current Cigarette Smoking Among Adults in the United States 2020. Available from: https://www.cdc.gov/tobacco/data_statistics/fact_sheets/adult_data/cig_smoking/index.htm.

[2] American Lung Association. Learn About COPD. 2021.

[3] Prochaska JJ, Das S, Young-Wolff KC (2017). Smoking, mental illness, and public health. Annu Rev Public Health.

[4] Yohannes AM, Hann M, Sibbald B (2011). The management of depressive symptoms in patients with COPD: a postal survey of general practitioners. Prim Health Care Res Devel.

[5] Pashutina Y, Kotz D, Kastaun S (2022). Attempts to quit smoking, use of smoking cessation methods, and associated characteristics among COPD patients. NPJ Prim Care Respir Med.

[6] Hurtado-Ruzza R, Álvarez-Calderón-Iglesias O, de Becerro-Bengoa-Vallejo R, Calvo-Lobo C, San-Antolín M, Losa-Iglesias ME (2022). Self-reported depression and anxiety among COPD patients. A case-control study. Sao Paulo Med J.

[7] Centers for Disease Control and Prevention. Smoking Cessation: Fast Facts. 2022.

[8] Richardson S, McNeill A, Brose LS (2019). Smoking and quitting behaviours by mental health conditions in Great Britain (1993–2014). Addict Behav.

[9] El-Mallakh P, McPeak D, Khara M, Okoli CT (2016). Smoking behaviors and medical Co-morbidities in patients with mental illnesses. Arch Psychiatr Nurs.

[10] Faul F, Erdfelder E, Lang A-G, Buchner A (2007). G*Power 3: a flexible statistical power analysis program for the social, behavioral, and biomedical sciences. Behav Res Methods.

[11] Faul F, Erdfelder E, Buchner A, Lang A-G (2009). Statistical power analyses using G*Power 3.1: tests for correlation and regression analyses. Behav Res Methods.

[12] Prochaska V (1997). The transtheoretical model of health behavior change. Am J Health Promot.

[13] Kroenke K, Spitzer RL, Williams JBW (2001). The PHQ-9: validity of a brief depression severity measure. J Gen Intern Med.

[14] Etter JF-o, Le Houezec J, Perneger TV (2003). A self-administered questionnaire to measure dependence on cigarettes: the cigarette dependence scale. Neuropsychopharmacology..

[15] Velicer WF, DiClemente CC, Prochaska JO, Brandenburg N (1985). Decisional balance measure for assessing and predicting smoking status. J Pers Soc Psychol.

[16] Prochaska V, DiClemente F (1988). Measuring processes of change: Applications to the cessation of smoking. J Consult Clin Psychol.

[17] American Lung Association. COPD Trends Brief 2020. Available from: https://www.lung.org/research/trends-in-lung-disease/copd-trends-brief.

[18] West Virginia Department of Health and Human Resources. Statistics about the Population of West Virginia 2021. Available from: https://dhhr.wv.gov/hpcd/data_reports/Pages/Fast-Facts.aspx.

[19] United States Census Bureau. Quick Facts West Virginia 2019. Available from: https://www.census.gov/quickfacts/fact/table/WV/INC910219#INC910219.

[20] United Health Foundation. 2021. Available from: https://www.americashealthrankings.org/explore/annual/measure/Depression_a/state/WV.

[21] Floyd J, Mallow J, Theeke L (2018). Health-related quality of life, depression, and smoking status in patients with COPD: results from the 2016 behavioral risk factor surveillance system data. Open J Nurs.

[22] Burns A, Strawbridge JD, Clancy L, Doyle F (2017). Exploring smoking, mental health and smoking-related disease in a nationally representative sample of older adults in Ireland - A retrospective secondary analysis. J Psychosom Res.

[23] Goodwin RD, Lavoie KL, Lemeshow AR, Jenkins E, Brown ES, Fedoronko DA (2012). Depression, anxiety, and COPD: the unexamined role of nicotine dependence. Nicotine Tob Res.

[24] Polit DF, Beck CT (2017). Nursing research: Generating and assessng evidence for nursing practice.

